# A Heavy Tailed Expectation Maximization Hidden Markov Random Field Model with Applications to Segmentation of MRI

**DOI:** 10.3389/fninf.2017.00066

**Published:** 2017-11-21

**Authors:** Diego Castillo-Barnes, Ignacio Peis, Francisco J. Martínez-Murcia, Fermín Segovia, Ignacio A. Illán, Juan M. Górriz, Javier Ramírez, Diego Salas-Gonzalez

**Affiliations:** ^1^Signal Processing and Biomedical Applications, University of Granada, Granada, Spain; ^2^Signal Processing Group, Carlos III University, Madrid, Spain; ^3^Department of Scientific Computing, Florida State University, Tallahassee, FL, United States; ^4^Department of Psychiatry, University of Cambridge, Cambridge, United Kingdom

**Keywords:** magnetic resonance image, brain tissue segmentation, gray matter, white matter, α-stable distribution, hidden Markov random fields

## Abstract

A wide range of segmentation approaches assumes that intensity histograms extracted from magnetic resonance images (MRI) have a distribution for each brain tissue that can be modeled by a Gaussian distribution or a mixture of them. Nevertheless, intensity histograms of White Matter and Gray Matter are not symmetric and they exhibit heavy tails. In this work, we present a hidden Markov random field model with expectation maximization (EM-HMRF) modeling the components using the α-stable distribution. The proposed model is a generalization of the widely used EM-HMRF algorithm with Gaussian distributions. We test the α-stable EM-HMRF model in synthetic data and brain MRI data. The proposed methodology presents two main advantages: Firstly, it is more robust to outliers. Secondly, we obtain similar results than using Gaussian when the Gaussian assumption holds. This approach is able to model the spatial dependence between neighboring voxels in tomographic brain MRI.

## 1. Introduction

The segmentation of brain MRI consist in the parcellation of the brain areas into their main tissue components: white matter (WM), gray matter (GM), and cerebrospinal fluid (CSF). Segmentation is an important preprocessing step in many brain image applications and it is currently an active field of research (Ortiz et al., 2012, 2014; Beare et al., 2016; Mulder et al., 2017; Serag et al., 2017).

A wide range of segmentation approaches assumes that the distribution of the histogram of intensity for each brain tissue can be modeled using a Gaussian distribution or a mixture of Gaussians (Laidlaw et al., 1998; Ruan et al., 2000). In Leemput et al. (1999) and Zhang et al. (2001), apart from the Gaussian assumption, spatial information is included in the mathematical model considering a hidden Markov random field.

Additional works using a Gaussian mixture model for describing the brain tissue histograms are Ashburner and Friston (2005), Greenspan et al. (2006), Ashburner and Friston (2007), and Merisaari et al. (2009). da Silva (2009) presents a Markov chain sampling technique for exploring normal mixture models when the numbers of components are unknown. In that work, a Gaussian mixture model with more than three components is used to explain the three brain tissues: GM, WM, and CSF.

Gaussian assumption and the hidden Markov random field model are currently being used as a segmentation procedure for magnetic resonance images (Xia et al., 2016; Liang et al., 2016; Nguyen et al., 2017; Wang et al., 2017). Nevertheless, this assumption presents some drawbacks that were pointed out in Salas-Gonzalez et al. (2013b). Assigning an unique Gaussian distribution to each brain tissue histogram is not accurate, as the histogram of white and gray matter exhibits heavy tails and certain degree of asymmetry. This problem is usually circumvented assigning more than one Gaussian distribution to each histogram, but, even using this approach, this procedure has also two additional drawbacks: Firstly, it is hard to identify the the boundary between the GM and WM histograms, specially when two small weighted Gaussian distributions are competing to explain data in that area. Secondly, a mixture of Gaussians does not exhibit heavy tails. In this work, we show that α-stable distributions are suitable to model the brain tissues more accurately.

A random variable with α-stable distribution fulfill the following property: a linear combination of two independent copies of the variable has the same distribution. Furthermore, the stable distributions are completely described by only four parameters [the characteristic exponent (α), degree of asymmetry (β), dispersion (γ) and location (δ)]. The Gaussian distribution is a particular case of α-stable distribution and, along with the Cauchy and Lévy distributions, they are the only α-stables probability density functions which can be written in closed form Samorodnitsky and Taqqu (1994). This is the reason why α-stable probability density functions (pdf) are evaluated numerically. These properties confer to the stable distribution the ability to fit asymmetric and heavy tailed data better than the Normal distribution (Salas-Gonzalez et al., 2009, 2010).

The α-stable distribution has been historically used in many fields of research (Physics, Economics or Engineering among others), but only recently it has been used for applications in brain image processing (Salas-Gonzalez et al., 2013a, 2014, 2015). In addition, the α-stable finite mixture model has been also used in the parametrization of GM and WM in MRI (Salas-Gonzalez et al., 2013b). In that work, it was stated that the α-stable distribution is able to fit the brain image tissues more accurately than the Gaussian distribution. Nevertheless, the finite mixture model performs an analysis of the histogram of intensity values, and it does not include spatial information in the model. Thus, that approach can only be used as a thresholding segmentation method.

In this article, we extend the work published in Salas-Gonzalez et al. (2013b). Specifically, we present a hidden Markov random field model with expectation maximization modeling the components using the α-stable distribution. As the Gaussian model is a particular limiting case of the α-stable distribution, the proposed model is a generalization of the widely used EM-HMRF assuming Gaussian distributions in the histogram of each component. The proposed methodology is tested in two datasets: synthetic data and a real problem of MRI segmentation of GM and WM.

This work is organized as follows: section 2 presents the hidden Markov random field algorithm proposed in this article along with the T1-weighted MRI database. The results are given and discussed in sections 3 and 4. Finally, the conclusions are drawn in section 5.

## 2. Materials and methods

### 2.1. Hidden Markov random field with α-stable distributions

The goal of the mathematical model is to assign each voxel in the image to one of the 2 components of the α-stable mixture model using a maximum a posteriori criterion. A similar model was presented in Wang (2012) assuming Gaussian distributions:

(1) $$\begin{array}{l} \hat{x}=\text{arg max}\{P(y|x)P(x)\}. \end{array}$$

***y*** is the 3D MRI image and ***x*** is a 3D matrix with same size as ***y*** containing information about the voxel assignment or allocation (*x*_*i*_ = 1 for GM and *x*_*i*_ = 2 for WM). In addition, *P*(***y***|***x***) is the likelihood probability of the observation and *P*(***x***) is the prior probability of the class (GM or WM).

*P*(***y***|***x***) is estimated assuming an α-stable distribution for the histogram of intensity values. As instance, let consider that a voxel *x*_*i*_ is allocated to GM (assigning *x*_*i*_ = 1) or WM (*x*_2_ = 2). Thus, the α-stable parameters of the GM histogram, can be calculated by selecting all the voxels that fulfill the condition *x*_*i*_ = 1 and performing likelihood estimation of the α-stable parameters (Nolan, 1997), obtaining θ_1_ = {α_1_, β_1_, γ_1_, δ_1_}. The same procedure is used for the estimation of the α-stable parameters of the WM histogram, which we denote as θ_2_ = {α_2_, β_2_, γ_2_, δ_2_}. The likelihood probability of the observation *P*(***y***|***x***) can be estimated straightforwardly. As instance, for a given intensity value *y*_*i*_ from the MRI brain image ***y***, the likelihood is *P*(*y*_*i*_|θ_1_) and *P*(*y*_*i*_|θ_2_). *P*(Δ|Δ) denotes the α-stable probability density function which is estimated numerically.

Under a Markov random field framework, these two probabilities are derived from:

(2) $$\begin{array}{l} P(x)=\frac{1}{Z}\exp(-U(x)) \end{array}$$

and

(3) $$\begin{array}{l} P(y|x)=\frac{1}{Z}\exp(-U(y|x)), \end{array}$$

where *Z* is a normalization constant (the partition function) and *U* denotes the energy function.

In this framework, we introduce spatial information in the model by means of the prior energy function *U*(*X*):

(4) $$\begin{array}{l} U(X)=\sum_{c\in C}V_{c}(X); \end{array}$$

where *V*_*c*_(***X***) is the clique potential and *C* is the set of all possible cliques (the 6 nearest voxels in the 3D space). The clique potential is defined on pairs of neighbors as:

(5) $$\begin{array}{l} V_{c}(x_{i},x_{j})=\beta_{C}(1-I_{x_{i},x_{j}}), \end{array}$$

with

(6) $$\begin{array}{l} I_{x_{i},x_{j}}=\{\begin{array}{ll} 0 & \text{if }x_{i}\neq x_{j}\\ 1 & \text{if }x_{i}=x_{j} \end{array}. \end{array}$$

β_*C*_ is a coefficient which models the contribution of the prior energy *U*(***X***) in the posterior energy term. We choose β_*C*_ = 0.5 as we obtain a good performance for the applications studied in this work using this value. Increasing this β_*C*_ parameter we increase the contribution of the neighboring voxels in the estimation of the class of a given voxel. In addition, the contribution of the pdf used for modeling the histogram data decreases when we increase the β_*C*_ parameter. Therefore, we would obtain the same accuracy values in the segmentation of images regardless of the chosen pdf, as only *U*(***X***) would contribute in the calculation of the posterior energy *U*(***X***|***Y***). On the other hand, in the limiting case when β_*C*_ = 0 the HMRF model transforms in the finite mixture model studied in Salas-Gonzalez et al. (2013b). We set *V*_*C*_ = 0 if a neighbor voxel belongs to a different tissue type and *V*_*C*_ = 1 if the class type of the voxels *x*_*i*_ and *x*_*j*_ are the same. This summation is taken over all 6 couples of voxels that are nearest neighbors in the image grid.

The joint likelihood of the model is

(7) $$\begin{array}{l} P(y|x)=\prod_{i\;=\;1}^{N}f_{\alpha_{i},\beta_{i}}(y_{i}|\gamma_{i},\mu_{i}) \end{array}$$

where *f*_α_*i*_, β_*i*__(*y*_*i*_|γ_*i*_, μ_*i*_) denotes an α-stable pdf which can be evaluated numerically. Parameter values depend on the allocation of variable *x*_*i*_. Therefore, we could also write: α_*i*_ = α_*x*_*i*__, β_*i*_ = β_*x*_*i*__, γ_*i*_ = α_*x*_*i*__, δ_*i*_ = δ_*x*_*i*__. In addition, as *Z* is a constant normalization term, the log-likelihood of the model is related to the likelihood energy

(8) $$\begin{array}{l} \log P(x|y)\propto -U(x|y), \end{array}$$

and, therefore, the maximum a posteriori estimation of the parameters is equivalent to the minimization of the posterior energy function.

#### 2.1.1. Expectation-maximization algorithm

The expectation-maximization approach is an iterative procedure for obtaining the unknown parameters of the model. This approach is performed in two differentiate steps. Firstly, we initialize the parameters of the data θ^(0)^, and then:

##### 1) Expectation

Let θ(*t*) be the current parameter values. We calculate the likelihood distribution for each of all possible labels (in this work *j* = 1, 2).

(9) $$\begin{array}{l} \prod_{i\;=\;1}^{N}w_{j}f_{\alpha_{j},\beta_{j}}(y_{i}|\gamma_{j},\delta_{j}) \end{array}$$

In addition, we estimate the weights of the model *w*_*j*_ as the proportion of the voxels allocated to component *j*.

##### 2) Maximization

We maximize the conditional expectation in order to update the parameter values:

(10) $$\begin{array}{l} \theta^{\left(t+1\right)}=\text{arg max}\left[\prod_{i\;=\;1}^{N}w_{j}f_{\alpha_{j},\beta_{j}}(y_{i}|\gamma_{j},\delta_{j})\right] \end{array}$$

Using the current values of the parameters, we calculate the allocation of variables $\hat{x}$ via a maximum a posteriori estimation.

(11) $$\hat{x}=\text{arg min}\{U(y|x)+U(x)\}.$$

Then, we set θ^(*t*+1)^ ← θ^(*t*)^ and repeat steps 1 and 2 until convergence.

### 2.2. MRI database

The set of 18 images used in this article were obtained from the Internet Brain Segmentation Repository^1^ (IBSR) which provides manually-guided expert segmentation results along with magnetic resonance brain image data.

The MRI image acquisition, according to the information provided by the Internet Brain Segmentation Repository documentation, follows this procedure: The coronal three-dimensional T1-weighted spoiled gradient echo MRI scans were performed on two different imaging systems. Ten FLASH scans on four males and six females were performed on a 1.5 tesla Siemens Magnetom MR System (Iselin, NJ) with the following parameters: TR = 40 ms, TE = 8 ms, flip angle = 50°, field of view = 30 cm, slice thickness = contiguous 3.1 mm, matrix = 256 × 256, and averages = 1. Ten 3D-CAPRY scans on six males and four females were performed on a 1.5 tesla General Electric Signa MR System (Milwaukee, WI), with the following parameters: TR = 50 ms, TE = 9 ms, flip angle = 50°, field of view = 24 cm, slice thickness = contiguous 3.0 mm, matrix = 256 × 256, and averages = 1.

Initially, we selected only the WM and GM voxels in the images. We studied the improvement of the hidden Markov random field model with α-stable distributions over the HMRF assuming Gaussian which is usually considered in the literature. Specifically, the goal of this study is to evaluate how this distribution assumption affects the segmentation in the boundary between GM and WM.

The HMRF model used for segmentation purposes requires the existence of a segmented image for the initialization of the algorithm. This initial segmented image is updated iteratively by the EM and the maximum a posteriori estimation steps using the hidden Markov random field algorithm presented in section 2.1. In this work, the initial segmented brain image ***x*** is built from the dataset using a leave-one-out procedure. As instance, for the image number *i*, we select the manually segmented images available in the IBSR dataset for all the images but the current image *i*. Then, we perform the affine registration of these segmented images to the current source image *i*. Finally, using the remaining 17 brain images, we build a tissue probability map in the spatial space of the image *i*. It is considered that a voxel initially belongs to a given tissue if allocation probability to this tissue is the maximum.

## 3. Results

### 3.1. Synthetic data

**Figure 1:**
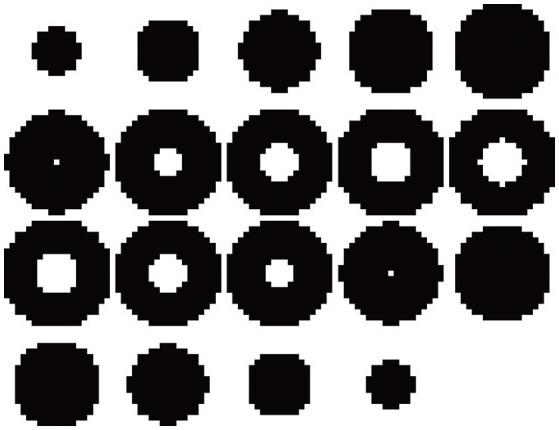
Montage showing the 20 slices with dimension 20 × 20 of the synthetic 3D source image.

**Figure 2:**
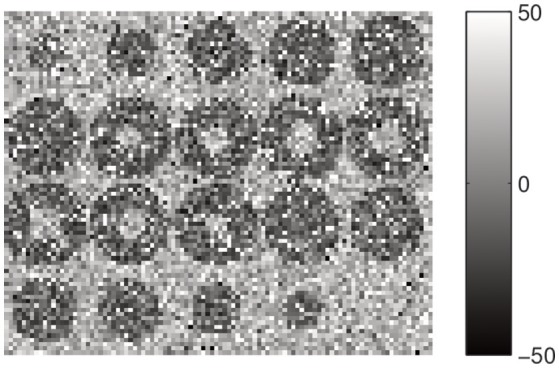
Montage showing the 20 slices with dimension 20 × 20 of the preliminar source image including the α-stable random noise.

We test the hidden Markov random field model to perform the segmentation of a synthetic 3D image with impulsive α-stable noise. This image is built as an empty sphere centered in a three-dimensional box with dimension 20 × 20 × 20 = 8,000 voxels. The background and inner sphere are built by a stable random distribution with parameters θ_1_ = {α_1_, β_1_, γ_1_, δ_1_} = 1.41100. The rest of the image is built using a random variable with θ_2_ = {1.801020}. The transaxial slices showing the preliminar position of the first and second component regions are plotted in Figure 1. In addition, Figure 2 shows a montage with all the slices of the source image, but, in that case the synthetic 3Dimage includes α-stable random noise. This synthetic data is very impulsive, as instance, their values range from −180 to 3,480. For the sake of clarity, we choose a grayscale colormap with −50 and 50 for white and black, respectively. Although there are 10 voxels with intensity values lower than −50 and 269 with intensity values greater than 50.

**Figure 3:**
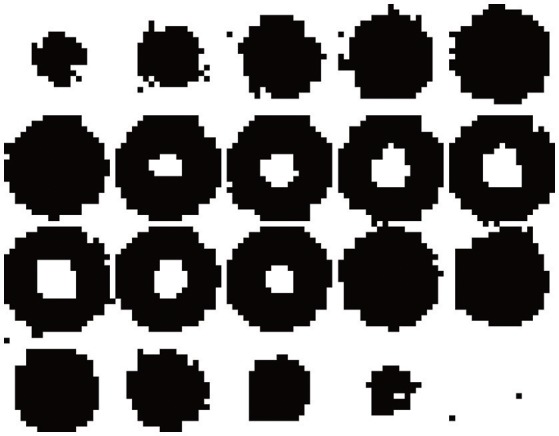
Montage showing the 20 slices with dimension 20 × 20 with the results of the segmentation.

**Figure 4:**
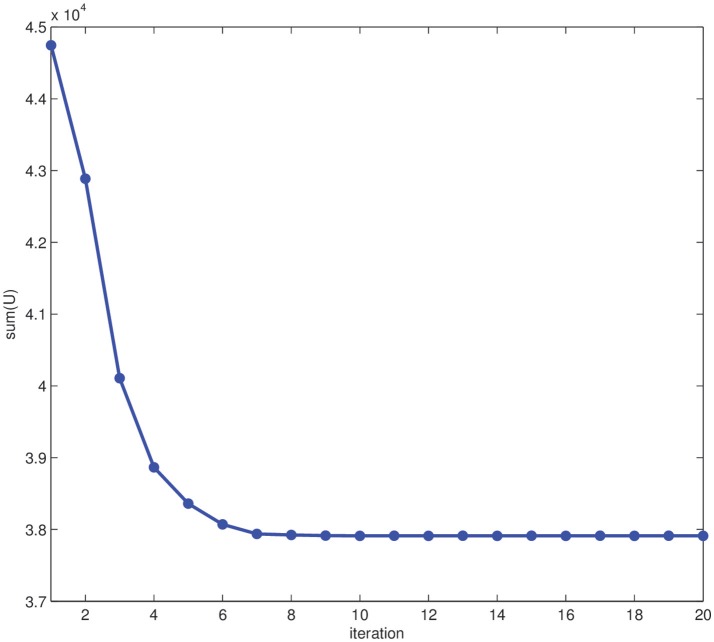
Sum of Log-Likelihood *U* in each iteration.

**Figure 5:**
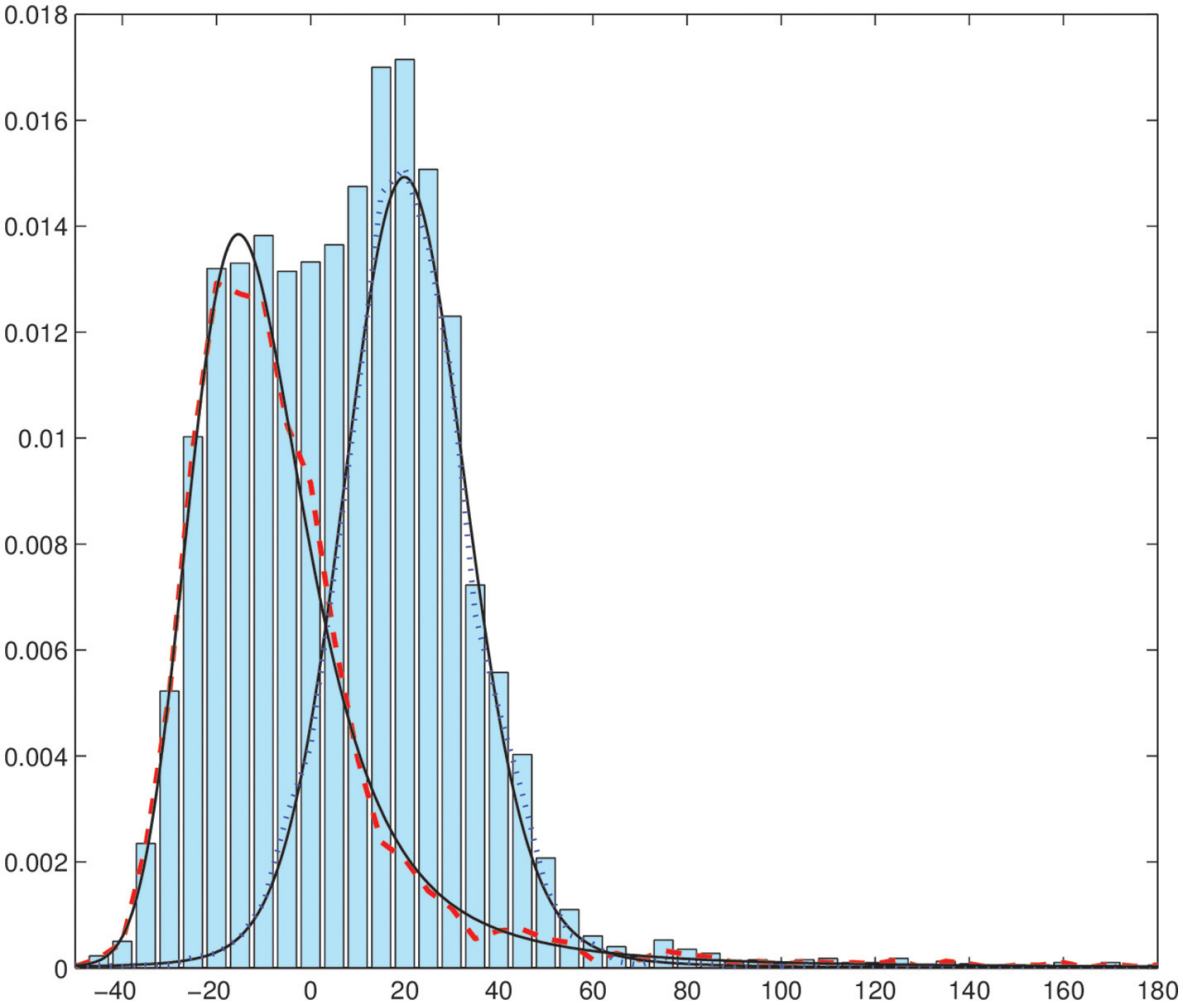
Histogram of voxel intensity values. Black continuous lines: predicted α-stable densities. Red dashed line, distribution of the estimated first α-stable component. Blue dotted line, distribution of the estimated second α-stable component.

We have performed the segmentation of this synthetic image in Figure 2 using the EM-HMRF model with α-stable distributions. Figure 3 shows the segmentation results after 20 iterations. The sum of the Log-Likelihood in each iteration when the α-stable model is used is depicted in Figure 4. As we can notice, convergency is reached after 7 iterations. The histogram of the voxel intensity values is also depicted in Figure 5 where the predicted α-stable density is plotted. The components of the mixture are very mixed and the overall histogram of intensity values seems clearly unimodal. Despite of that fact, predicted densities fit very accurately the truth distributions of the two regions .

**Figure 6:**
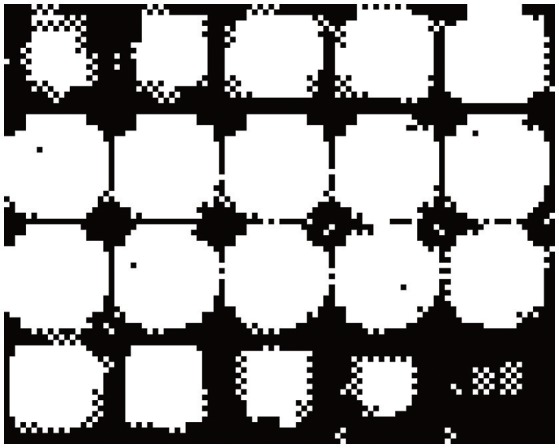
Montage showing the 20 slices with dimension 20 × 20 with the results of the segmentation when the 2-component EM-HMRF with Gaussian distributions is used.

**Figure 7:**
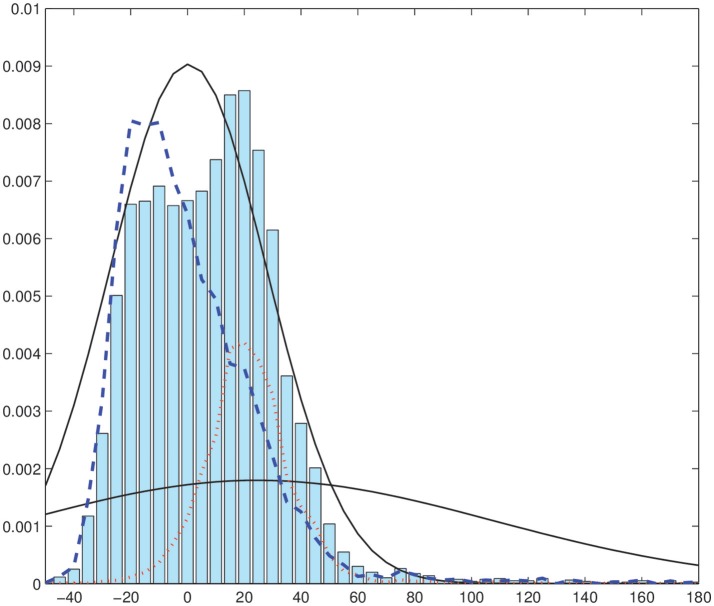
Histogram of voxel intensity values. Black continuous lines, predicted Gaussian densities. Red dashed line, distribution of the estimated first Gaussian component. Blue dotted line, distribution of the estimated second Gaussian component.

We have performed the same analysis and segmentation procedure but considering a EM-HMRF with Gaussian distributions. Figure 6 shows the results when the Gaussian model is considered. In that case, Gaussian assumption does not lead to satisfactory results due to the presence of outliers in the model. Specifically, one component tries to fit the bulk of the distribution modeling most of the voxels in the image, while the second component, which has a very large variance, tries to model the outliers in the image. In addition, we obtain 96% of accuracy when the α-stable EM-HMRF is used and 85% with the Gaussian. Mainly, the Gaussian model fails in the segmentation of the inner part of region 1. This behavior is highlighted in Figure 7, where the predicted 2-mixture Gaussians densities are plotted.

**Figure 8:**
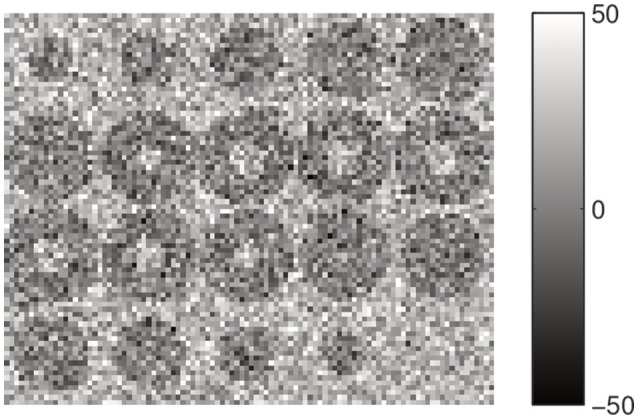
Montage showing the 20 slices with dimension 20 × 20 of the synthetic source image including the Gaussian random noise.

One of the main advantages of the α-stable distribution upon the Gaussian is that the later is a particular case of stable distribution. Therefore, the approach presented in this paper can be viewed as a generalization of the EM-HMRF with Gaussian distributions. In order to test the flexibility of this proposed model and its ability to work in Gaussian noise environments, we have tested the performance of our method by segmenting synthetic data with additive Gaussian noise. For that reason, we repeat the previous analysis but using a synthetic 3D image with Gaussian noise. Again, the image is an empty 3D sphere centered in a box with dimension 20^3^ = 8,000 voxels. The outer and inner region is built by a Gaussian random variable with μ_1_ = 0 and σ_1_ = 14.14 while the region number two is randomly sampled from a Gaussian with values μ_2_ = 20 and σ_2_ = 14.14. Figure 8 shows a montage with all the slices of the source image. The distribution of this synthetic image is formed by a mixture of two Gaussian components and their values range from −49.4 to 67.1.

We obtained the same accuracy in the segmentation results (95.4% of voxels correctly identified) using the α-stable or Gaussian model. Specifically, we got the following parameter values for the distribution of the first and second region: θ_1_ = {2, −9.62, −0.15} and θ_2_ = {2, −9.60, 20.8}. As a result of this experiment, we have proved that Gaussian parameter values were estimated accurately, as the α-stable dispersion parameter and the Gaussian standard deviation σ are related with the formula $\sigma =\sqrt{2}\gamma$. The β parameter is undefined when α = 2.

**Figure 9:**
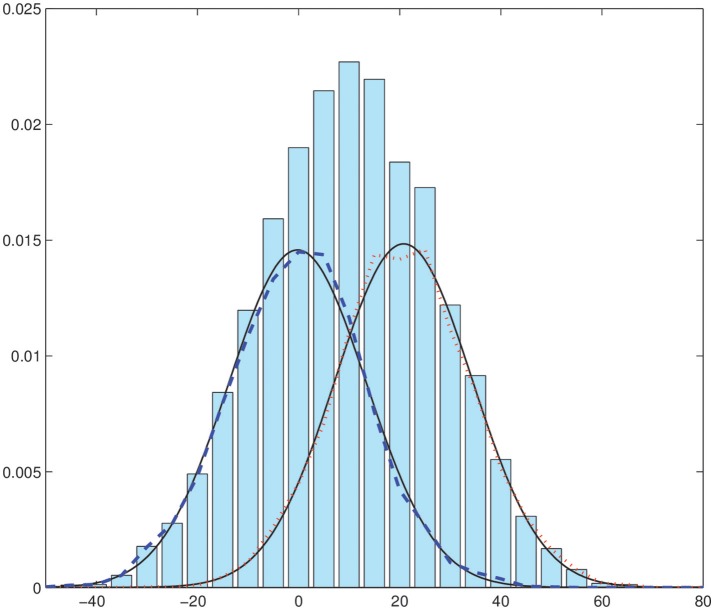
Histogram of voxel intensity values. Black continuous lines, predicted α-stable densities. Red dashed line, distribution of the estimated first α-stable component. Blue dotted line, distribution of the estimated second α-stable component.

Histogram of voxel intensity values and the predicted density are plotted in Figure 9. Again, as in the first example, the two original components are quite mixed.

### 3.2. MRI data

Recently, we have published a study of the histogram of GM and WM intensity values (Salas-Gonzalez et al., 2013b). There, we restricted our study to the global analysis of histograms and we did not include any spatial information in the model. In this work, we show how the HMRF can be used to enhance this previous work. Thus, the proposed α-stable methodology is used to include spatial information in the model by means of the neighboring relations between voxels. This information can be used to perform the segmentation of gray matter and white matter tissues in brain magnetic resonance images. We test the EM-HMRF α-stable algorithm in a dataset of 18 magnetic resonance images from the Internet Brain Segmentation Repository and compare the proposed methodology with respect to the Gaussian assumption.

Table 1 shows the accuracy segmentation results obtained using the EM-HMRF α-stable model. We compare these results with the assumption of a Gaussian distribution for the GM and WM histograms (Zhang et al., 2001).

**Table 1:** MRI accuracy segmentation results and Dice similarity coefficient.

**Accuracy α-stable**	**Accuracy Gaussian**	**GM Dice α-stable**	**GM Dice Gaussian**	**WM Dice α-stable**	**WM Dice Gaussian**
**0.9010**	0.8926	0.8472	0.8287	0.9266	0.9263
0.9168	**0.9195**	0.8988	0.8931	0.9202	0.9281
**0.8945**	0.8936	0.8318	0.8299	0.9287	0.9287
**0.9161**	0.9125	0.8713	0.8613	0.9386	0.9396
**0.9003**	0.8901	0.8461	0.8217	0.9242	0.9266
**0.9155**	0.8658	0.8694	0.7812	0.9300	0.9261
**0.9196**	0.9140	0.8697	0.8543	0.9393	0.9430
**0.9141**	0.8809	0.8401	0.7886	0.9443	0.9342
**0.8934**	0.8905	0.8273	0.8216	0.9295	0.9297
**0.9186**	0.9004	0.8451	0.8125	0.9462	0.9441
**0.9119**	0.8993	0.8539	0.8332	0.9416	0.9386
**0.9154**	0.8932	0.8380	0.8014	0.9471	0.9408
**0.8903**	0.8899	0.8136	0.8120	0.9285	0.9291
0.9253	**0.9260**	0.9029	0.9019	0.9324	0.9339
0.9185	**0.9233**	0.9179	0.9133	0.9140	0.9243
0.8788	**0.8811**	0.8435	0.8525	0.8987	0.8960
**0.9104**	0.9085	0.8876	0.8975	0.9185	0.9096
**0.8733**	0.8728	0.8581	0.8586	0.8795	0.8777

*Stable, proposed EM-HMRF α-stable mixture model. Gaussian, EM-HMRF model with Gaussian distributions. Bold: Best accuracy results*.

**Figure 10:**
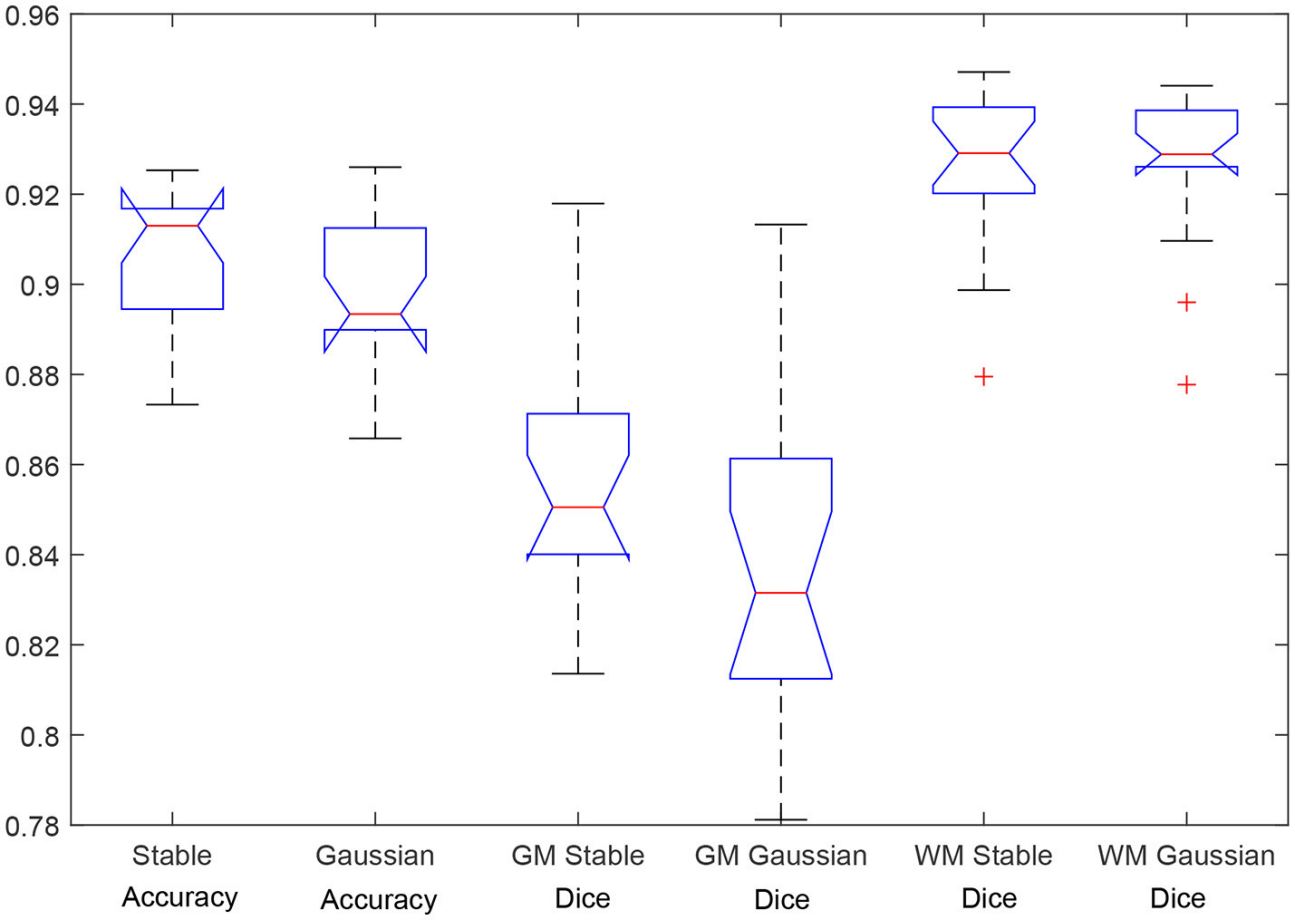
Segmentation results: accuracy and Dice similarity coefficient.

Figure 10 shows a box plot with the accuracy results and the Dice similarity coefficient in Table 1. As usual, the central mark is the median, and the edges of the box are the 25th and 75th percentiles. The bars extend to the most extreme data points. This plot shows that the HMRF α-stable algorithm performs better than Gaussian version of the algorithm. Specifically, the following median accuracy values are obtained: 0.9141 (α-stable EM-HMRF) and 0.8936 (Gaussian EM-HMRF).

**Figure 11:**
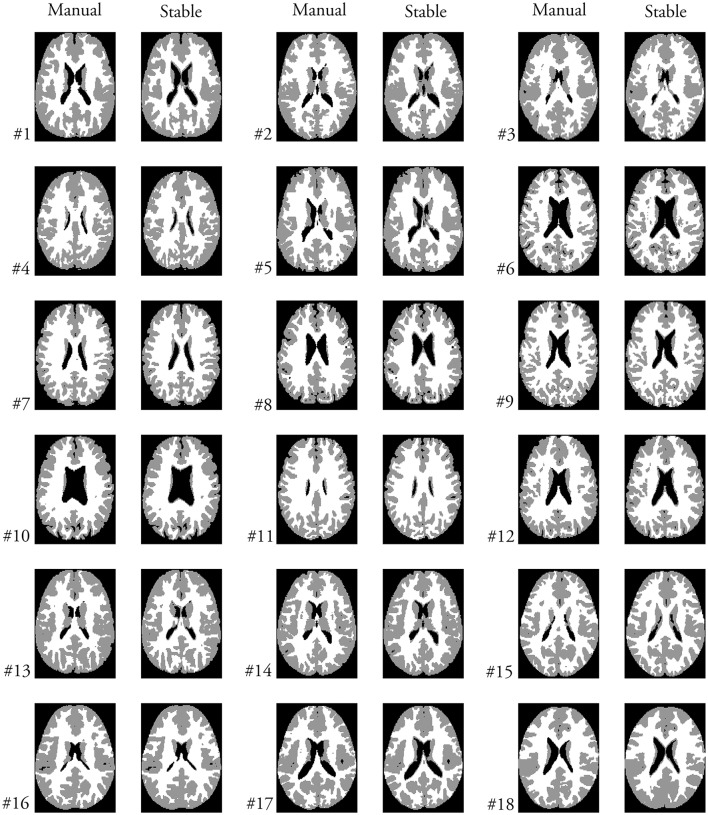
A selected transaxial slice displaying the ground truth segmentation (Manual) and the Stable segmentation.

Figure 11 shows the manual segmentation (Manual) considered the ground truth and the segmentation results (Stable) in a transaxial slice for each of the 18 brain images from the IBSR dataset. This figure allows us to visually inspect the performance of the heavy tailed HMRF segmentation method.

## 4. Discussion

The method presented in this work, along with the previous work in parameterization of the brain tissues can be used to develop novel brain image processing methods under a heavy-tailed assumption. Let note that, in this paper, we do not focus on building a complete segmentation methods with several different preprocessing steps or parameters to calibrate. Instead of that, we study and isolate one common assumption which is made by many segmentation approaches in the literature, as instance: the Gaussian distribution can be used to fit the histogram of intensity values of the GM and WM tissue in MRI. We showed in this work that Some advantages of the α-stable EM-HMRF method can be summarized as follows:

- It allows us to model each histogram of brain tissues using only one distribution.
- It allows us to deal with heavy-tailed data.
- It allows to fit asymmetric distribution in a parsimonious way.
- As the Gaussian is a limiting case of the α-stable distribution, we expect to obtain the same results when the Gaussian assumption is correct. This was found to be the case when we applied the α-stable EM-HMRF to synthetic data.

### 4.1. Future work

- The α-stable HMRF approach opens a potential direction to develop novel applications in neuroimage in the future. As instante, the applications of the mathematical model presented here is not limited to the segmentation of magnetic resonance images. Other image modalities with heavy-tailed distribution can also benefit from the algorithm presented here, as instance FP-CIT SPECT images used for Parkinson's disease diagnosis were found to present also heavy tails (Salas-Gonzalez et al., 2013a).
- An additional step will be to extend the algorithm in order to include CSF in the model. Nevertheless, the histogram of their intensity values are not heavy-tailed, therefore, a different strategy should be envisaged for this tissue.

## 5. Conclusion

In this work, we have presented an expectation maximization hidden Markov random field algorithm for the segmentation of white and gray matter in magnetic resonance images. In order to achieve this goal, distributions of intensity values of GM and WM have been modeled using α-stable distributions. This method has been tested using synthetic and real brain MRI data. We have compared the proposed methodology to the modelization of these tissue distributions using Gaussians. We have obtained a better performance in terms of accuracy when the α-stable model is used.

## Author contributions

All authors listed have made a substantial, direct and intellectual contribution to the work, and approved it for publication.

### Conflict of interest statement

The authors declare that the research was conducted in the absence of any commercial or financial relationships that could be construed as a potential conflict of interest.

## Footnotes

^1^http://www.cma.mgh.harvard.edu/ibsr
